# Exploration of the relationships between clinical traits and functional connectivity based on surface morphology abnormalities in bulimia nervosa

**DOI:** 10.1002/brb3.2930

**Published:** 2023-03-15

**Authors:** Weihua Li, Miao Wang, Guowei Wu, Jiani Wang, Xiaohong Li, Zemei Yang, Qian Chen, Zhenghan Yang, Zhanjiang Li, Peng Zhang, Lirong Tang, Zhenchang Wang

**Affiliations:** ^1^ Department of Radiology, Beijing Friendship Hospital Capital Medical University Beijing China; ^2^ Chinese Institute for Brain Research Beijing China; ^3^ CAS Key Laboratory of Behavioral Science Institute of Psychology, Chinese Academy of Sciences Beijing China; ^4^ The National Clinical Research Center for Mental Disorders & Beijing Key Laboratory of Mental Disorders, Beijing Anding Hospital Capital Medical University Beijing China

**Keywords:** eating disorders, surface‐based functional connectivity, surface‐based morphometry

## Abstract

**Background:**

Bulimia nervosa is a recurrent eating disorder with uncertain pathogenesis. Recently, there has been growing interest in using neuroimaging techniques to explore brain structural and functional alterations in bulimia nervosa, but the findings of previous studies have a great number of inconsistencies.

**Methods:**

Here, we collected anatomical and resting‐state functional magnetic resonance imaging data from 43 bulimia nervosa patients and 34 matched healthy controls (HCs). We applied a surface‐based morphology analysis to explore brain cortical morphology differences and a novel surface‐based functional connectivity (FC) analysis to investigate functional abnormalities. Principal component analysis was performed to analyze the behavioral data of the participants. We further analyzed the relationships between abnormalities in cortical characteristics or FC and clinical features.

**Results:**

We observed increased greater sulcal depth in the right superior temporal gyrus (STG) and the right medial orbitofrontal cortex (mOFC) in bulimia nervosa patients than in the HCs. Additionally, the patients exhibited increased FC between the right STG and right ventral tegmental area but decreased function between the right mOFC and right putamen, which was significantly negatively correlated with the first principal component reflecting the severity of bulimia nervosa symptom.

**Conclusions:**

Our findings provide evidence of neuroanatomical and functional abnormalities in bulimia nervosa patients. Moreover, the FC between the right mOFC and right putamen was associated with symptom severity of bulimia nervosa, which may be a neural marker and involved in the neuropathological mechanism of bulimia nervosa.

## Graphical abstract

We conducted a novel surface‐based FC analysis based on surface morphology alterations in BN, PCA to analyze the behavioral data of the participants and further explored the relationships between neuroimaging findings and principal component. Altered FC based on surface morphology alterations was related with related to the severity of BN symptom. Our findings provided further evidence that reward processing and body image processing may be involved in BN.

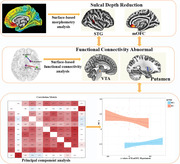

## 1 INTRODUCTION

Bulimia nervosa (BN) is a complex psychiatric and psychological disorder with a typical onset in adolescence or early adulthood and primarily affects females, and long‐term patients can experience serious medical complications affecting the whole‐body system. The cardinal symptoms of BN are recurrent episodes of binge eating followed by self‐induced vomiting or another inappropriate compensatory behavior and negative self‐evaluation of body shape and weight (American Psychiatric Association, 2013). Clinical treatment of BN remains a significant challenge, with only less than half of the patients exhibiting prolonged remission (Hagan & Walsh, 2021). A better understanding of neurobiological processes involved in BN will facilitate the development of new interventions.

Previous neuroimaging studies have provided supporting evidence demonstrating that BN is related to brain structural and functional changes. For example, previous studies adopting voxel‐based morphology (VBM) analyses have associated BN with altered gray matter (GM) volume (GMV) in the medial orbitofrontal cortex (mOFC), striatum, and insula, which are related to processing reward information and/or self‐regulation (Frank et al., 2013; Schafer et al., 2010). Another study found reduced GMV in the superior frontal gyrus, superior temporal gyrus (STG), and cingulate and paracingulate gyri, which may be associated with impaired inhibitory control and self‐body dissatisfaction (Li et al., 2020). These studies revealed correlations between BN and brain structures to some extent; however, these studies adopted VBM analyses, which are thought to neglect the intrinsic geometry of the highly folded human cortex. Surface‐based morphology (SBM) analyses provide a better alignment of cortical landmarks to explore whether structural variability on the cortical surface is related to BN. Some brain structural studies using SBM analyses have found cortical abnormalities in BN, such as reduced cortical thickness, volumetric reductions in local brain regions, and localized deformations on the surface of subcortical structures, but concluded that there is a high level of heterogeneity and inconsistency across these studies (Berner et al., 2018, 2019, 2018, 2019; Marsh et al., 2015; Westwater et al., 2018). In addition, few investigations have used a combined strategy to assess the differences in cortical morphology and associated functional connectivity (FC) differences in BN patients compared to healthy controls (HCs). An algorithm for surface‐based FC was developed to calculate vertex‐wise FC between regions of interest (ROIs) and to compare the differences between BN patients and HCs (Brodoehl et al., 2020). The algorithm proposed an inflated original cerebral surface map of neuronal activity in the sulci and on the gyri, which also avoided disturbances from white matter (WM) and cerebrospinal fluid (CSF) signals. Moreover, the algorithm reduced the false positives of activation around the boundaries of adjacent but functionally separated brain regions, which were probably identified in the volume‐based analyses.

Thus, in the present research, we conducted an SBM analysis and examined surface‐based FC analysis to further illustrate the functional changes based on neuroanatomical variations and to understand how brain areas interact with one another. The purpose was to clarify the cortical morphology abnormalities in BN patients, and we further hypothesized that the FC abnormalities would emerge in the regions identified in the regional SBM analysis, according to the previous neuroimaging studies of BN. In exploratory analyses, we also assessed whether correlations existed between clinical traits and possible FC differences based on surface morphology abnormalities in BN.

## 2 MATERIALS AND METHODS

### 2.1 Participants

A total of 43 patients diagnosed with BN and 34 HCs, comparable in age, sex, handedness, and educational level, participated in this study. Patients were recruited in the outpatient clinic department of the Eating Disorders Unit in the hospital. We recruited HCs with normal weight and without any history of mental illness via the internet. All study subjects were right‐handed. The BN patients were clinically diagnosed by two clinical psychiatrists, one of whom was an expert in eating disorders, according to the Diagnostic and Statistical Manual of Mental Disorders, 5th Edition (DSM‐5) criteria for BN. We used the Mini‐International Neuropsychiatric Interview to exclude subjects with any potential psychiatric illness among the HCs (Sheehan et al., 1998). Among the 43 patients, 9 had a history of anorexia nervosa and 12 had a history of antidepressant medication, but none of them have used any psychotropic drugs within the last 2 months before the MRI examination. Of those patients, six have depressive symptoms, eight have anxiety symptoms, and six have obsessive–compulsive symptoms. Specific exclusion criteria were currently diagnosed with other severe psychiatric disorders, including bipolar disorder, schizophrenia, dissociative disorder, substance abuse disorder within the last year, and so on; the previous history of brain surgery and neurologic disorders; pervasive developmental disorder and intellectual disability; the history of significant health problems, such as endocrine disorders (including diabetes, hyperlipidemia, and hyperthyroidism); claustrophobia, pregnancy, lactation, and metallic implants (including pacemakers and nonremovable metal dental retainers).

This study was approved by the Institutional Review Board of the Beijing Friendship hospital (the approval number is 2021‐P2‐052‐01) and performed in accordance with the Declaration of Helsinki ethical standards. All subjects provided written informed consent.

### 2.2 Behavioral assessments

Before arrival at the scanning visit, all participants were asked to fast for at least 4 h. All participants were asked to complete several self‐report questionnaires before the MRI acquisition scan. The participants rated their current hunger intensity using a visual analog scale (VAS) scored from 0 indicating “not at all” to 10 indicating “extremely.” The study participants completed the Chinese version of the Dutch Eating Behavior Questionnaire (DEBQ) consisting of restrained, emotional, and external eating subscales to assess their eating behavior (Wu et al., 2017). A total of 33 items of the DEBQ are completed on a 5‐point scale that ranges from 1 “never” to 5 “very often.” The Eating Disorder Inventory‐I (EDI‐I) and Eating Attitudes Test (EAT‐26) were used to measure participants’ symptoms and concerns characteristic of eating disorders (Kang et al., 2017; Lee et al., 1997). We assessed depressive and anxiety symptoms using the Beck Depression Inventory‐II (BDI‐II) and self‐rating anxiety scale (SAS), respectively (Shek, 1990; Zung, 1971). The Chinese version of these scales has good reliability and validity and can be used as effective tools for the evaluation of eating disorders among Chinese people (Kang et al., 2017; Lee et al., 1997; Mo et al., 2020; Shek, 1990; Wu et al., 2017; Zung, 1971).

### 2.3 MRI data acquisition

All participants were scanned on a Siemens Prisma 3.0 T MRI system (Erlangen, Germany) equipped with a 64‐channel head coil. We used a conventional axial T2 sequence to exclude participants with any possible brain abnormalities. High‐resolution anatomical MRI datasets with 192 1.0 mm contiguous sagittal slices for all participants were acquired using a T1‐weighted, three‐dimensional, magnetization‐prepared rapid gradient‐echo sequence (echo time [TE], 2.98 ms; repetition time [TR], 2530 ms; inversion time, 1100 ms; flip angle [FA], 7°; bandwidth, 240 Hz/Px; isotropic voxel size, 1 × 1 × 1 mm^3^; data matrix, 256 × 256; and field of view [FOV], 256 × 256 mm^2^). The resting‐state functional MRI images were obtained using an echo‐planar imaging sequence (TE, 30 ms; TR, 2000 ms; FA, 90°; bandwidth, 2368 Hz/Px; FOV, 224 × 224 mm^2^; data matrix, 64 × 64; number of slices, 33; slice thickness, 3.5 mm; interslice gap, 1 mm; and total volumes, 240).

To reduce imaging noise and minimize head motion, we used tight but comfortable earplugs and foam padding around the subject's head throughout the entire 16‐min scanning process (8 min for resting‐state scan). The participants were instructed to rest with their eyes closed but stay awake and avoid specific thoughts. After each sequence scan, we confirmed the participant's status, and all reported that they had not fallen asleep during the entire scanning process.

### 2.4 Anatomical and resting‐state functional data preprocessing

The structural image preprocessing used the computational anatomy toolbox (CAT12, http://www.neuro.uni‐jena.de/cat/) that was implemented in the Statistical Parametric Mapping analysis package (SPM12 version, www.fil.ion.ucl.ac.uk/spm/software/spm12/). Steps included (1) denoising using spatially adaptive nonlocal means filter; (2) tissue segmentation for CSF, WM, and GM volumetric structures; (3) spherical correction of the GM–WM interface and the GM–CSF interface where triangular mesh tessellation was adapted to smoothly and perfectly represent WM surface and pial surface; (4) spatial normalization transforming individual native space to fsaverage stereotaxic space; (5) deformation estimation and the registration from an inflated spherical mesh of individual brain to common spherical coordinate system (HCP fsLR32k).

The functional image preprocessing implementing fMRIprep (https://fmriprep.org/en/stable/)(Esteban et al., 2019) and xcp‐Engine (https://github.com/PennLINC/xcpEngine)(Ciric et al., 2018) included the following steps: (1) motion correction using the flirt function of FSL; (2) slice timing correction for each volume; (3) 4D global mean‐based intensity normalization; (4) coregistration of individual structural and functional brain images by the boundary‐based registration algorithm of FreeSurfer; (5) projection of individual preprocessed rsfMRI images of each time point to the standard cortical surface (fsaverage5); (6) transformation of images from fsaverage5 space to HCP fsLR32k space for higher resolution and a symmetric left‐to‐right parcellation; (7) interpolation of intensity outliers in each vertex's time series using AFNI's 3dDespike utility, demeaning and removal of any linear or quadratic trends was further applied; (8) nuisance regression with 36 regressors from fMRIPrep outputs after filtering and excluding outliers (framewise displacement [FD] > 0.3). Regressors included 6 head motion measures, the global signal, the mean WM signal, the mean CSF signal, and the temporal derivatives of these 12 parameters, as well as 18 quadratic expansions of 6 motion measures, 3 signals, and 9 derivatives (Ciric et al., 2017, 2018); (9) bandpass filtering (0.01–0.08 Hz) was further applied to residual time series from the regression; (10) finally, the data were smoothed with the Connectome Workbench and a kernel size of 6.0 mm. Given the intergroup differences in movement parameters that can confound resting‐state fMRI results, we compare the FD of movement parameters between the groups and found no significant differences between groups (*t* = −.85, degrees of freedom = 68, and *p* = .396). For the detailed preprocessing steps for fMRI data, please refer to Supporting Information 1.

### 2.5 Data analysis

#### 2.5.1 Clinical data analysis

Four HCs were excluded due to nonremovable metal dental retainers and claustrophobia during fMRI scanning, and one patient and two HCs were excluded due to FD > 0.2; none was excluded for neuroanatomical abnormality; a total of 42 BN patients (2 males) and 28 HCs (2 males) were finally included in the analysis. The baseline demographic information and clinical characteristics were analyzed using SPSS 25.0 software. Between‐group comparisons were performed using independent two‐sample *t*‐tests or chi‐square tests, as appropriate. *p* < .05 was considered statistically significant. Continuous variables are presented as the mean with 95% confidence intervals.

Additionally, we used principal component analysis (PCA), a dimensionality‐reduction method for related multivariate analyses, to analyze the behavioral data of the participants. PCA was performed to explore the degree of association and capture the commonality purely within the behavioral questionnaires. More specifically, the nine scores from the seven questionnaires, including the VAS, number of binge‐eating times per week, DEBQ, EDI‐BN, EAT, BDI, and SAS, were included in the PCA. The components with a high ratio of variance and eigenvalue greater than 1 were reserved in the PCA (Boehmke & Greenwell, 2019).

#### 2.5.2 SBM analysis

SBM analyses of cortical indicators (thickness, surface area, volume, mean curvature, and sulcal depth) were automatically performed using CAT 12 to measure different properties of brain cortical surface morphology (Luders et al., 2006). Cortical thickness is the averaged linking distance between the pial and WM surfaces along the normal vector. Surface area refers to each vertex as the average area of the triangles that surround it. Volume is defined as the product of cortical thickness and surface area. The curvature at each vertex is calculated by averaging the maximum and minimum bending of the cortical surface. Sulcal depth is defined as the product of the displacement and regular unit vector in the process of the expansion of the cortex and is used for quantifying the large‐scale geometric information of the cortex. Finally, 22‐mm full width at half maximum smoothing to the resampled surface data for all metrics was applied. A two‐sample *t* test and a cluster‐based familywise error (FWE) correction with *k* = 144 and *p* < .05 were further applied to the results of all metrics with age controlled for through CAT12.

#### 2.5.3 Calculation of surface‐based functional connectivity

ROIs identified in the SBM analysis were used as seeds to compute FC with each vertex on the surface and voxel in subcortical regions through the Pearson correlations. A Fisher‐*z* transformation was used for subsequent statistics. Surfstat was conducted to investigate the intergroup differences between the BNs and HCs by a two‐sample *t*‐test with the regression of age, educational level, and mean FD values (Worsley et al., 2009). The results of surface‐based FC were reserved through cluster‐based false discovery rate (FDR) (uncorrected vertex wise *p* < .001, cluster wise *p* < .05) correction in Surfstat, and voxel‐based subcortical FC was corrected with voxel‐wise FDR (*p* < .05) correction in MATLAB, respectively.

#### 2.5.4 Correlation analyses between clinical variables and both SBM and FC values

The correlation analysis was subsequently conducted to test associations between behaviors and both FC and SBM metrics by a multiple variable linear regression model (lm function in R), with the covariates age and sex controlled. Group information was also included in regression models to test the interaction effect between brain features and group categories for BNs or HCs. First, we construct full models that take the FC and SBM results (along with covariates) as independent variables. The four best models were separately determined by the backward solution method implemented in the step function of the stats package for PC1‐FC, PC1‐SBM, PC2‐FC, and PC2‐SBM. Finally, only models of PC1‐FC and PC1‐SBM were reserved after the Bonferroni correction (please refer to Supporting Information 2 for details). In addition, we further tested whether a correlation existed between SBM findings and the corresponding FC in the BN group through linear regression.

## 3 RESULTS

### 3.1 Baseline and behavioral characteristics

Table 1 shows the descriptive statistics for the demographic and behavioral data of all participants. The results were considered significantly different at the level of *p* < .05 (two‐tailed), and the two groups did not differ for the demographic data (sex, age, BMI, and fasting hours). The EAT‐26, EDI‐BN, DEBQ, BDI, and SAS scores in the BN group were higher than those in the HC group.

**TABLE 1:** Baseline demographics and clinical characteristics

	BN patients (*n* = 42)		HCs (*n* = 28)		
Characteristics	Mean	SD	Mean	SD	*p*
Sex (F/M)	40/2		26/2		.728^a^
Age (years)	24.67	5.98	25.93	2.85	.242^b^
BMI (kg/m^2^)	21.40	3.84	20.89	2.06	.479^b^
Fasting hours (h)	9.65	4.09	8.43	4.01	.220^b^
Educational level (years)	16.42	1.901	17.40	2.45	.097^b^
Age of illness onset (years)	21.10	5.09			
Illness duration (months)	42.14	48.28	–	–	–
Binge/purging frequency (times/week)	5.98	4.40	–	–	–
EAT‐26	43.95	11.55	11.82	8.28	.000^b^ ^,c^
EDI‐BN	33.14	6.40	11.75	3.76	.000^b^, ^c^
DEBQ—total	121.21	15.29	84.96	14.73	.000^b^, ^c^
DEBQ—restrained eating	38.69	6.16	27.68	6.54	.000^b^, ^c^
DEBQ—emotional eating	47.19	12.32	26.29	10.18	.000^b^, ^c^
DEBQ—external eating	35.21	6.47	31.00	4.78	.003^b^, ^c^
BDI	23.38	8.64	4.31	6.39	.000^b^, ^c^
SAS	54.18	12.16	33.35	8.28	.000^b^, ^c^

Abbreviations: BDI, Beck Depression Inventory; BMI, body mass index; BN, bulimia nervosa; DEBQ, Dutch Eating Behavior Questionnaire; EAT, Eating Attitudes Test; EDI, Eating Disorder Inventory; HCs, healthy controls; SAS, self‐rating anxiety scale; SD, standard deviation.

^a^ *p* Values were obtained by chi‐square tests.

^b^ *p* Values were obtained by two‐sample *t*‐tests.

^c^ For all tests, *p* < .05 was considered statistically significant.

**FIGURE 1:**
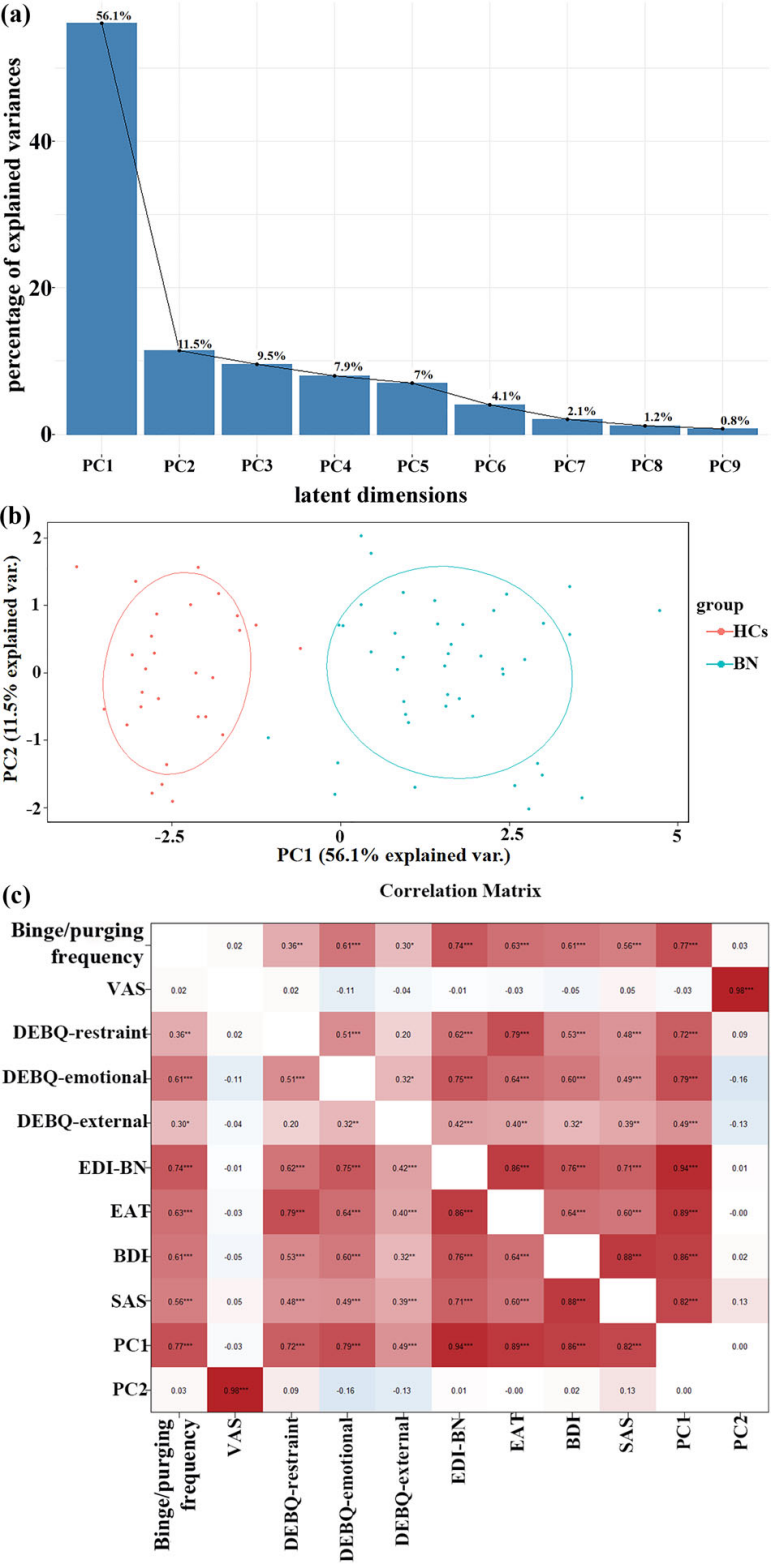
Part (a) shows latent dimensions of behavioral traits in the bulimia nervosa (BN) group. Part (b) shows a loadings plot (correlation scaled) for principal component analysis (PCA) of eating behavior and psychological trait questionnaire scale scores: Principal component (PC)1 denotes the first principal component that explained 56.1% of the sample variance, and PC2 denotes the second component that explained 11.5% of the sample variance. Part (c) shows the correlation analysis matrix of the PCA components and all questionnaire scores. PC1 showed strong correlations (*p* > .7) with emotion‐related symptoms and binge‐eating frequency, whereas the PC2 was correlated with visual analog scale (VAS) scores. BDI, Beck Depression Inventory; DEBQ, Dutch Eating Behavior Questionnaire; EAT, Eating Attitudes Test; EDI, Eating Disorder Inventory; SAS, self‐rating anxiety scale.

PCA identified that the first two principal components were significant, with *R* ^2^ values of .561 and .115, hence cumulatively explaining 67.6% of the variation in the dataset (see Figure 1a). Figure 1b shows the two‐dimensional loadings plot (correlation scaled). The first component (PC1) showed strong correlations (range *r* = .72–.94) with emotion‐related symptoms and binge/purging frequency, including binge/purging times per week and DEBQ‐emotional, DEBQ‐restraint, EDI‐BN, EAT, BDI, and SAS scores, whereas the second principal component (PC2) was correlated with VAS scores (*r* = .98) (see Figure 1c).

### 3.2 SBM results

**FIGURE 2:**
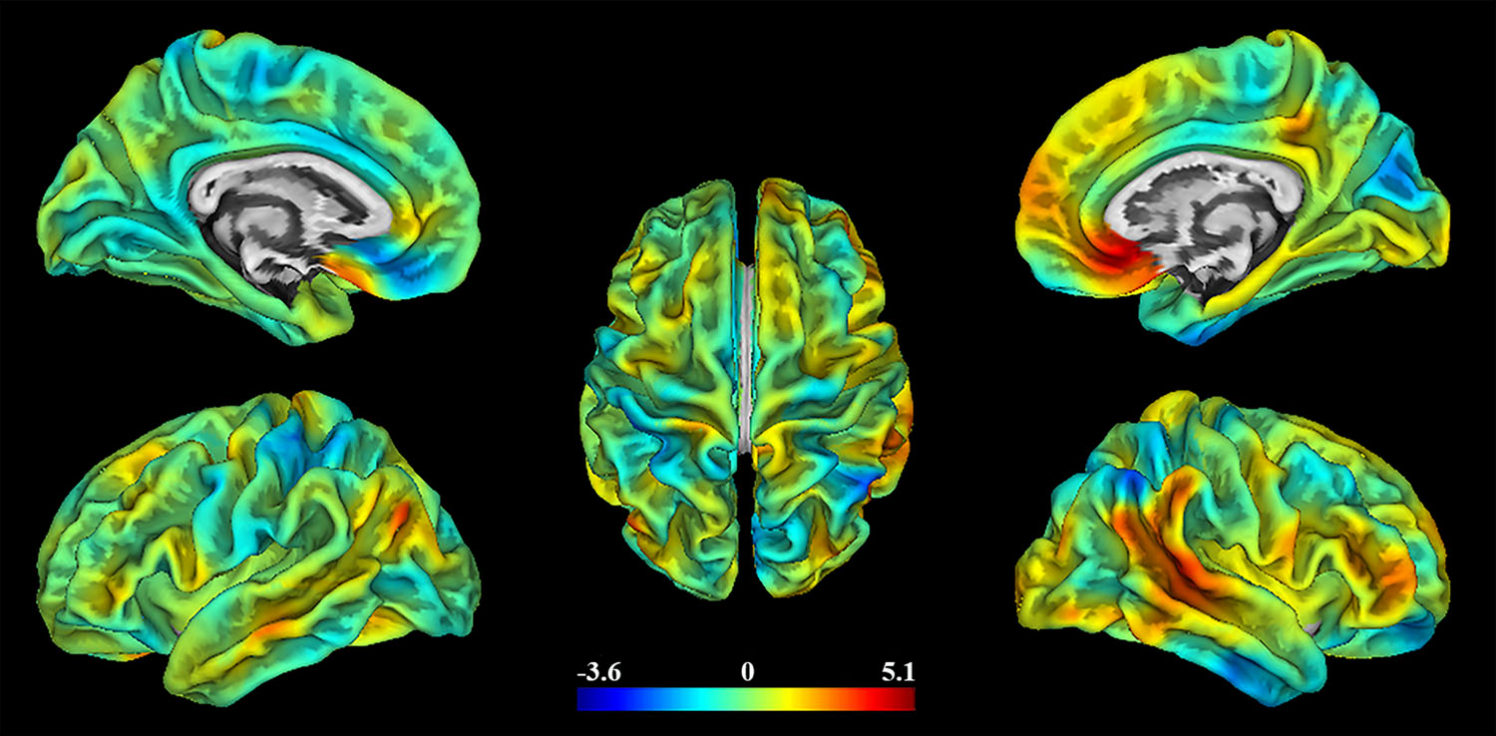
A pseudocolor map of the *t* value distribution across the whole brain, which represents the group differences in sulcal depth; the color bar indicates the voxel‐wise *t*‐values.

**FIGURE 3:**
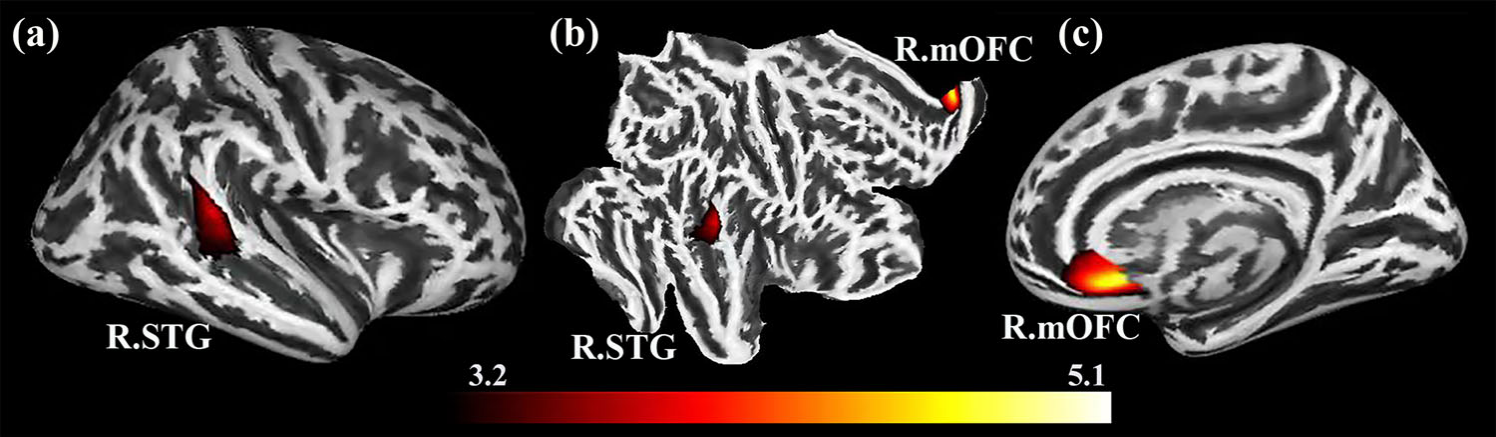
(a–c) Clusters that significantly varied in terms of sulcal depth in the bulimia nervosa (BN) group versus the healthy control (HC) group. The patients with BN had a significantly greater sulcal depth in the right superior temporal gyrus (STG) and right medial orbitofrontal cortex (mOFC) than the HCs (familywise error [FWE] correction, *p* < .05). The color bar indicates the voxel‐wise *t*‐values.

There were no significant between‐group differences in cortical thickness, surface area, volume, or mean curvature. Figure 2 shows a pseudocolor map of the *t*‐value distribution across the whole brain, which represents the group differences in sulcal depth. The patients with BN displayed significantly greater sulcal depth values in the right STG (*t* = 5.13, *p* = .001) and right mOFC (*t* = 4.0, *p* = .039) than the HCs (corrected with FWE, *p* < .05; but the right mOFC failed to be corrected by more rigorous Bonferroni correction, *p* < .01) (see Figure 3). Table 2 shows the cluster sizes and peak values in the two brain regions.

**TABLE 2:** Between‐group difference on sulcal depth

*p* Value at peak	Cluster size (no. of vertex)	Hemisphere	Overlap with DK40 atlas
.00008	337	R	76% bankssts 15% superior temporal 7% supramarginal
.00000	155	R	52% medial orbitofrontal 46% rostral anterior cingulate

*Note*: Significance threshold was set at *p* < .05 corrected with FWE at the cluster level, and the results were reported based on the DK40 atlas.

Abbreviations: DK40 atlas, Desikan–Killiany 40 atlas; FEW, familywise error; R, right.

### 3.3 Group differences in surface‐based FC between the BN group and HC group

**FIGURE 4:**
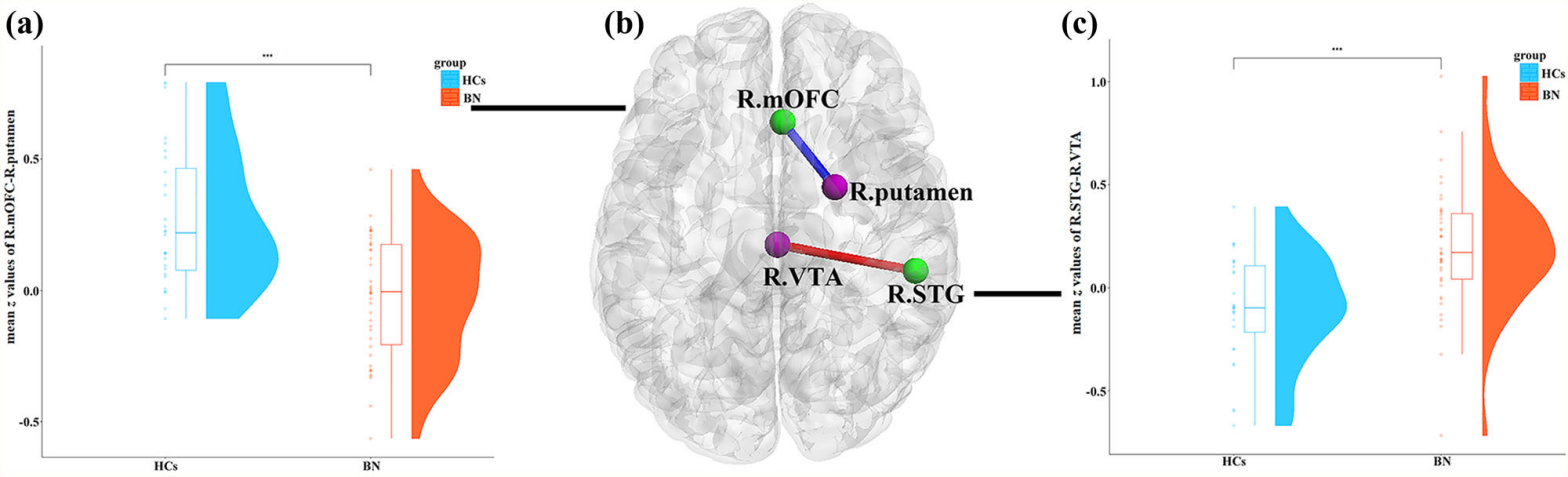
(a and c) Raincloud plots show the intergroup mean *z* values of functional connectivity (FC) between the right medial orbitofrontal cortex (mOFC) and right putamen and between the right superior temporal gyrus (STG) and right ventral tegmental area (VTA). (b) (Axial views) shows the intergroup differences in FC between the right mOFC and STG. The blue line illustrates the decreased FC between the right mOFC and right putamen (bulimia nervosa [BN] < healthy controls [HCs]). The red line illustrates the increased FC between the right STG and right VTA (BN > HCs). The results were corrected at *p* < .05 with false discovery rate (FDR) correction.

The FC results revealed that, compared with the HCs, the BN group exhibited increased FC between the right STG and right ventral tegmental area (VTA) but decreased FC between the right OFC and right putamen (see Table 3, Figure 4).

**TABLE 3:** Intergroup functional connectivity (FC) differences for the right medial orbitofrontal cortex (mOFC) and superior temporal gyrus (STG)

				MNI coordinates		
Seeds	Brain regions	Cluster size (voxels)	Peak *t*‐values	*x*	*y*	*z*
R.STG	R.VTA	13	−4.4766	2	−20	−8
R.mOFC	R.putamen	60	4.7788	24	2	0

*Note*: Results were corrected *p* < .05 with FDR.

Abbreviations: FDR, false discovery rate; MNI, Montreal Neurological Institute; R, right; VTA, ventral tegmental area.

### 3.4 Correlation analyses between clinical variables and SBM results and FC values

**FIGURE 5:**
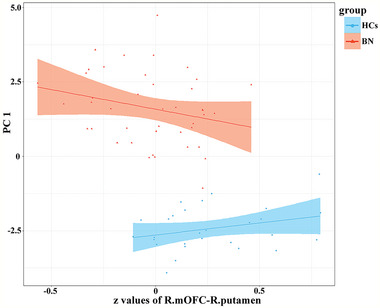
The bulimia nervosa (BN) group exhibited a significant negative correlation between principal component (PC)1 and functional connectivity (FC), between the right medial orbitofrontal cortex (mOFC) and right putamen (*r* = −.302, *p* = .019), whereas the healthy control (HC) group showed a positive correlation (not significant, *r* = .160, *p* = .223).

In the FC‐behavior correlation analysis, the general linear regression model of PC1‐FC was reserved with *F* = 26.69 (*p* < .001) and adjusted *R* ^2^ = .804. A significant interaction effect was found, as PC1 was significantly negatively correlated with FC between the right mOFC and right putamen in the BN group (*r* = −.302, *p* = .019), but not in the HC group (not significant, *r* = .160, *p* = .223) (see Table 4, Figure 5). In addition, we performed moderation analysis with the interaction package to assess the effect of PC1 (independent variable) on FC (dependent variable) in the BN and HC group. We found the interaction effect on PC1 between group and FC is significant. There is a significant negative correlation between FC of the right mOFC‐right putamen and PC1 on the BN group but not significant on the HC group (see Table 4). Furthermore, there were no significant correlations between the increased sulcal depth and their corresponding FC in the BN group.

**TABLE 4:** The general linear regression model of PC1‐functional connectivity (FC)

								Moderation analysis	
group	FC	*β*	Std. E	*t*	*p*	(95% CI of *β*)	*r* (partial)	*t*	*p*
BN	R.mOFC/R.putamen	−.194	.080	−2.412	.019	(−.354, −.033)	−.302	−2.024	.047
HC	R.mOFC/R.putamen	.114	.092	1.232	.223	(−.071, .299)	.160	1.110	.271

*Note*: *F* (11,58) = 26.69, *p* < .001, *R* ^2^ = .835 (adjusted *R* ^2^ = .804), *N* = 70.

Abbreviations: β, standardized coefficients; BN, bulimia nervosa; CI, confidence interval; HC, healthy control; mOFC, medial orbitofrontal cortex; Std. E, standard deviation error.

## 4 DISCUSSION

In this study, we demonstrated that, compared with HCs, BN patients displayed increased sulcal depth in the right mOFC and right STG. We further analyzed the surface‐based FC differences of the right mOFC and right STG to understand how the brain areas interact with one another. The present results revealed that the BN group exhibited increased FC between the right STG and right VTA but decreased FC between the right mOFC and the right putamen. However, there were no significant correlations between the increased sulcal depth and their corresponding FC in the patients with BN. For the brain and behavioral correlation analysis, the PC1 component performed significantly negatively correlated with FC between the right mOFC and right putamen in the BN group but showed a positive correlation in the HC group.

Increased sulcal depths in the right mOFC and STG were found in our study, but the increased sulcal depths were not significantly correlated with the corresponding FC in the BN group. Our findings expand the current understanding of structural alterations implicating the OFC and STG in BN (Frank et al., 2013; Li et al., 2020; Schafer et al., 2010; Westwater et al., 2018). However, we did not find morphological changes in cortical thickness, surface area, or subcortical shape abnormalities as did previous SBM studies, which might be due to sample limitations, including course, the severity of disease, and other related factors (Berner et al., 2018, 2019; Marsh et al., 2015). Sulcal depth is an important marker of brain anatomy in neuroscience, which can provide the information about the shape of cortical surface and reflect cortical folding features (Im et al., 2008; Lyu et al., 2018). Therefore, the increased sulcal depth in the BN group may be a sensitive surface parameter and related to disease development to some extent in BN. To date, the cause for the increased sulcal depth in the right mOFC and STG in BN remains unclear, and the underlying mechanisms are likely to be very complex. One potential explanation is the effects of long‐term recurrent binge eating/purging in the BN groups, which need to be tested further.

The mOFC has been associated with food avoidance and plays an important role in food intake control and satiety (Monteleone et al., 2018; Plassmann et al., 2010; Rolls, 2008). Previous VBM studies reported increased GMVs in the mOFC and striatum in BN patients compared with HCs (Frank et al., 2013; Schafer et al., 2010). Some fMRI findings demonstrated hypoactivity in the mOFC in BN patients when viewing and tasting food cues, and this attenuated response was related to the frequency of binge‐eating/purging episodes in BN (Frank et al., 2011; Uher, et al., 2004). It is likely that structural and functional changes in the OFC directly contribute to the occurrence and maintenance of BN. In our study, decreased FC between the right putamen and right mOFC was observed in the BN patients. The dorsal striatum, including the putamen and caudate nucleus is one of the important brain regions involved in the regulation of the brain reward system. The results of a previous VBM study suggested that brain structural alterations in the mOFC and dorsal striatum in BN were involved in the brain circuity assessing reward value, and alterations in the putamen were correlated with sensitivity to reward (Frank et al., 2013). A recent resting‐state fMRI study also reported disrupted resting‐state FC of putamen subregions in BN (Wang et al., 2020). Another event‐related fMRI study, involving the performance of a no‐go task, found that BN patients showed decreased activation in the right dorsal striatum and the right sensorimotor area, including the post‐ and precentral gyri (Skunde et al., 2016). Taken together, brain structure in the right mOFC and associated FC are altered in BN and suggest altered brain circuitry that has been associated with reward value. Moreover, we found that reduced FC between the right mOFC and right putamen was linked to symptom severity in the patients with BN, which is partly supported by previous findings. For example, several systematic reviews have indicated that illness severity of BN was associated with diminished activation in frontostriatal circuits (Donnelly et al., 2018; Frank, 2015; Steward et al., 2018). Specifically, in our study, the PC1 component corresponded to emotion‐related symptoms and binge‐eating frequency, which reflects the symptom severity of BN and could distinguish BN patients from HCs. The FC‐behavioral regression analysis revealed a significant interaction effect between the PC1 component and the FC between the right mOFC and right putamen in the BN and HC groups, that is, the PC1 component was significantly negatively correlated with the FC values in the BN group but showed a positive correlation in the HC group. That is, the higher the FC value between the right mOFC and right putamen in the BN patients, the lower the symptom severity, and the closer the FC value of the BN patients was to the HC group, the milder the symptom severity. These findings support the notion that reward‐processing deficits are associated with symptom severity in BN.

In addition, our study also found increased FC between the right STG and right VTA in the BN group. The STG, an area where the temporal and parietal lobes meet and are responsible for face processing and social cognition, has been shown to be associated with BN. The STG, incorporating information from the limbic system and somatosensory systems, plays a key role in social perception. Some studies have revealed that the smaller volume of the STG in BN may cause abnormal body image perception and excessive focus on body weight and shape, leading to binge‐eating/purging behavior (Li et al., 2020; Solstrand Dahlberg et al., 2017). Another structural MRI study showed that the STG was associated with body dissatisfaction in Japanese individuals (Kohmura et al., 2017). The VTA of the midbrain, which is rich in dopaminergic neurons, is the key brain region of the mesocorticolimbic pathway and is involved in the pathophysiology of BN (Jiang et al., 2019). The increased FC between the right STG and right VTA may indicate the aberrant reward‐based body image processing underlying in the STG.

Some limitations should be noted in this study. Our sample size was modest, and the results should be verified and extended with much larger samples. In addition, some patients in our study had mild anxiety or depressive symptoms that may have confounded our findings, but this may also make the sample more representative of the typical BN population (Godart et al., 2015). The cross‐sectional nature of our study means that further work is needed to clarify whether the observed cortical morphological abnormalities and related FC changes are the results of BN pathology or the consequence of binge‐eating and compensatory behaviors.

## 5 CONCLUSION

Our findings suggest that increased sulcal depth in the right mOFC and STG was observed in BN patients compared to HCs, and FC changes in the regions may be related to aberrant reward processing and body image processing, which may contribute to the development and persistence of BN. We further found that the morphological abnormality in the right mOFC and reduced FC with the right putamen may play a contributory role in the psychopathology of BN.

## CONFLICT OF INTEREST STATEMENT

The authors declare that there is no conflict of interest that could be perceived as prejudicing the impartiality of the research reported.

### PEER REVIEW

The peer review history for this article is available at https://publons.com/publon/10.1002/brb3.2930.

## Supporting information

Supporting Information

## Data Availability

The datasets involved in this study are available from the corresponding author on the reasonable request.
