# Supplementary material for: Exploration of the relationships between clinical traits and functional connectivity based on surface morphology abnormalities in bulimia nervosa
Source: Brain Behav. 2023 Mar 15;13(4):e2930. doi: 10.1002/brb3.2930 (PMC10097070; doi:10.1002/brb3.2930)
Supplement: Supplementary file 1 — Supporting Information [file BRB3-13-e2930-s001.docx]

**Supplementary material**

**Supplement 1：Preprocess of fMRI data**

First, we used custom methodology in fMRIprep to generate a reference volume and its skull-stripped version. According to fMRIPrep’s fieldmap-less approach, a deformation field to correct for susceptibility distortions was estimated. The deformation field is that resulting from coregistering the blood oxygen level-dependent (BOLD) signal reference to the same-subject T1w reference with its intensity inverted^[1, 2]^. Registration was conducted with antsRegistration (ANTs 2.3.3), and the process was regularized by constraining deformation to be nonzero only along the phase-encoding direction and modulated with an average fieldmap template^[3]^. Based on the estimated susceptibility distortion, a corrected echo-planar imaging (EPI) reference was calculated to be more accurately coregistered with the anatomical reference. Then, the BOLD reference was coregistered to the T1w reference using bbregister (FreeSurfer), which implements boundary-based registration^[4]^. Coregistration was configured with six degrees of freedom. Before any spatiotemporal filtering using mcflirt (FSL 5.0.9), head-motion parameters with respect to the BOLD reference (transformation matrices and six corresponding rotation and translation parameters) were estimated^[5]^. BOLD runs were slice-time corrected with 3dTshift from AFNI 20160207 (RRID:SCR_005927)^[6]^. The BOLD time series were then resampled onto the following surfaces (FreeSurfer reconstruction nomenclature): fsaverage by using mri_vol2surf (FreeSurfer). Then, the highest resolution fsaverage was used as an intermediate standardized surface space to generate Grayordinates files^[7]^ containing 91k samples for each subject.

Several confounding time series were calculated based on the preprocessed BOLD signals, including framewise displacement (FD) and three regionwise global signals. FD was calculated following power (the absolute sum of relative motions)^[8]^. The three global signals were extracted from the CSF, WM, and whole-brain masks. Outputs from fMRIPrep^[9]^ were postprocessed using the XCP-D^[10, 11]^. For each of the 1 CIFTI runs per subject, the following postprocessing step was performed: before nuisance regression and filtering, any volumes with FD greater than 0.3 were flagged as outliers and excluded from nuisance regression^[8, 10, 12]^. A total of 36 nuisance regressors were screened from the nuisance confound matrices of the fMRIPrep output. These nuisance regressors included six motion parameters: the mean white matter, mean CSF signal with their temporal derivatives, global signal, quadratic expansion of six motion parameters, tissue signals and their derivatives^[10, 11]^. We used the linear regression in Scikit-Learn 0.23.1 to regress these nuisance regressors from the BOLD data^[13]^. Then, residual time series from this regression were bandpass filtered within the frequency band of 0.01-0.08 Hz.

**References**

[1] HUNTENBURG J, GORGOLEWSKI K, ANWANDER A, et al. Evaluating nonlinear coregistration of BOLD EPI and T1 images [M]. 2014.

[2] WANG S, PETERSON D J, GATENBY J C, et al. Evaluation of Field Map and Nonlinear Registration Methods for Correction of Susceptibility Artifacts in Diffusion MRI [J]. Front Neuroinform, 2017, 11(17.

[3] TREIBER J M, WHITE N S, STEED T C, et al. Characterization and Correction of Geometric Distortions in 814 Diffusion Weighted Images [J]. PLoS One, 2016, 11(3): e0152472.

[4] GREVE D N, FISCHL B. Accurate and robust brain image alignment using boundary-based registration [J]. Neuroimage, 2009, 48(1): 63-72.

[5] JENKINSON M, BANNISTER P, BRADY M, et al. Improved optimization for the robust and accurate linear registration and motion correction of brain images [J]. Neuroimage, 2002, 17(2): 825-841.

[6] COX R W, HYDE J S. Software tools for analysis and visualization of fMRI data [J]. NMR Biomed, 1997, 10(4-5): 171-178.

[7] GLASSER M F, SOTIROPOULOS S N, WILSON J A, et al. The minimal preprocessing pipelines for the Human Connectome Project [J]. Neuroimage, 2013, 80(105-124.

[8] POWER J D, MITRA A, LAUMANN T O, et al. Methods to detect, characterize, and remove motion artifact in resting state fMRI [J]. Neuroimage, 2014, 84(320-341.

[9] ESTEBAN O, MARKIEWICZ C J, BLAIR R W, et al. fMRIPrep: a robust preprocessing pipeline for functional MRI [J]. Nat Methods, 2019, 16(1): 111-116.

[10] SATTERTHWAITE T D, ELLIOTT M A, GERRATY R T, et al. An improved framework for confound regression and filtering for control of motion artifact in the preprocessing of resting-state functional connectivity data [J]. Neuroimage, 2013, 64(240-256.

[11] CIRIC R, ROSEN A F G, ERUS G, et al. Mitigating head motion artifact in functional connectivity MRI [J]. Nat Protoc, 2018, 13(12): 2801-2826.

[12] SATTERTHWAITE T D, WOLF D H, RUPAREL K, et al. Heterogeneous impact of motion on fundamental patterns of developmental changes in functional connectivity during youth [J]. Neuroimage, 2013, 83(45-57.

[13] PEDREGOSA F, VAROQUAUX G, GRAMFORT A, et al. Scikit-learn: Machine Learning in Python [J]. J. Mach. Learn. Res., 2011, 12(2825–2830.

**Supplement 2：Regression model**

The prediction of PC using brain imaging features with significant intergroup differences by a multiple variable liner regression model (lm function in R) was conducted to test the associations between behaviors and both FC and SBM metrics, with the covariates age and sex controlled for. Group information was also included in regression models to test the interaction effect between brain features and group category for BNs or HCs. The four best models were separately determined by the backward solution method implemented in the step function of the stats package for PC1-FC, PC1-SBM, PC2-FC and PC2-SBM. The general linear regression models of PC1-FC and PC1-SBM fit with F=26.69 (p＜0.001), adjusted R^2^=0.804 and F=37.66 (p＜0.001), adjusted R^2^=0.788, respectively. The general linear regression model of PC2-FC and PC2-SBM fit with F=1.45 (p=0.174), adjusted R^2^=0.068 and F=1.40 (p=0.223), adjusted R^2^=0.039, respectively. Finally, only the PC1-FC and PC1-SBM models were reserved after Bonferroni correction. The general linear regression model of PC1-SBM was attached below (**see Table 5**).

**Table 5. The general linear regression model of PC1-SBM**

| group | brain regions | β | Std.E | t | p | [95% CI of β] | r(partial) |
| --- | --- | --- | --- | --- | --- | --- | --- |
| HC | R.STG | -0.023 | 0.087 | -0.269 | 0.789 | [-0.198, 0.151] | -0.034 |
|  | R.mOFC | 0.084 | 0.090 | 0.937 | 0.352 | [-0.095, 0.263] | 0.118 |
| BN | R.STG | -0.522 | 0.451 | -1.158 | 0.251 | [-1.424, 0.379] | -0.146 |
|  | R.mOFC | -0.017 | 0.445 | -0.038 | 0.970 | [-0.906, 0.872] | -0.005 |

*F (7,62) =37.66, p < 0.001, R^2^= 0.810 (Adjusted R^2^=0.788), N= 70*

*β, Standardized coefficients; Std.E, Standard Deviation Error; CI, confidence interval*

And the full model is as follows.

library(bruceR)

NEWS: A new version of bruceR (version 0.8.9) is available on 2022-08-11!
 Please update:
 install.packages("bruceR")
 update.packages(ask=FALSE)

bruceR (version 0.8.7)
 BRoadly Useful Convenient and Efficient R functions

 Packages also loaded:
 √ dplyr √ emmeans √ ggplot2
 √ tidyr √ effectsize √ ggtext
 √ stringr √ performance √ cowplot
 √ forcats √ lmerTest √ see
 √ data.table

 Main functions of `bruceR`:
 cc() Describe() TTEST()
 add() Freq() MANOVA()
 .mean() Corr() EMMEANS()
 set.wd() Alpha() PROCESS()
 import() EFA() model_summary()
 print_table() CFA() lavaan_summary()

 https://psychbruce.github.io/bruceR/

data <- import("E:/code/behavior_fc_vbm.xlsx")

√ Successfully imported: 29 obs. of 26 variables

setwd("E:/code/all_papers_data/SBMfc_data")
depth_data <- import("final_depth_project.xlsx")

New names:
 • `name` -> `name...3`
 • `name` -> `name...14`

√ Successfully imported: 70 obs. of 20 variables

TTEST(depth_data,"rmsd.y","group")

Independent-Samples t-test

 Hypothesis: two-sided (μ2 - μ1 ≠ 0)

 Descriptives:
 ─────────────────────────────────────
 Variable Factor Level N Mean (S.D.)
 ─────────────────────────────────────
 rmsd.y group CON 28 0.11 (0.03)
 rmsd.y group SUB 42 0.10 (0.03)
 ─────────────────────────────────────

 Levene’s test for homogeneity of variance:
 ───────────────────────────────────────────────────────
 Levene’s F df1 df2 p
 ───────────────────────────────────────────────────────
 rmsd.y: group (SUB - CON) 0.79 1 68 .378
 ───────────────────────────────────────────────────────
 Note: H0 = equal variance (homoscedasticity).
 If significant (violation of the assumption),
 then you should better set `var.equal=FALSE`.

 Results of t-test:
 ──────────────────────────────────────────────────────────────────────────────────────────────
 t df p Difference [95% CI] Cohen’s d [95% CI] BF10
 ──────────────────────────────────────────────────────────────────────────────────────────────
 rmsd.y: group (SUB - CON) -0.85 68 .396 -0.01 [-0.02, 0.01] -0.21 [-0.70, 0.28] 3.41e-01
 ──────────────────────────────────────────────────────────────────────────────────────────────

depth_mean <- import('depth_mean.csv')

√ Successfully imported: 74 obs. of 4 variables

depth_data <- depth_mean %>% dplyr::rename(ID=subname) %>%inner_join(depth_data,by=c("group","ID"))

all_data <- import("patient_all_info.xlsx")

√ Successfully imported: 89 obs. of 28 variables

depth_data %>% inner_join(all_data,by=c("ID","tall",'weight','group')) -> all_info_data
all_info_data$overeatting_time_perweek <- as.numeric(all_info_data$overeatting_time_perweek)

all_sub_pca <- all_info_data %>% dplyr::select(overeatting_time_perweek,VAS,DEBQ_33_Restraint,DEBQ_33_emotion,DEBQ_33_external,
 EDI_BN,EAT_26,BDI_21,SAS_20)%>% na.omit() %>% prcomp(center = TRUE,scale. = TRUE)

library(bruceR)
all_info_data %>% dplyr::select(sig_stat_data_PUTAMEN_R,sig_stat_data_DIENCEPHALON_VENTRAL_R,
 data_cluster1,data_cluster2,sig_seed1_mean_dep,sig_seed2_mean_dep,
 overeatting_time_perweek,group,age,sex.x)%>%na.omit()%>%
 cbind(all_sub_pca$x[,1:2]) -> new_data

pc1_model <- lm(PC1~sig_stat_data_PUTAMEN_R*group+sig_stat_data_DIENCEPHALON_VENTRAL_R*group+
 data_cluster1*group+data_cluster2*group+age+sex.x,data=new_data)
GLM_summary(pc1_model)

General Linear Model (OLS Regression)

 Model Fit:
 F(11, 58) = 26.69, p = 1e-18 ***
 R² = 0.83502 (Adjusted R² = 0.80373)

 Unstandardized Coefficients:
 Outcome Variable: PC1
 N = 70
 ───────────────────────────────────────────────────────────────────────────────────────────────────────
 b S.E. t p [95% CI of b] VIF
 ───────────────────────────────────────────────────────────────────────────────────────────────────────
 (Intercept) -2.391 (2.103) -1.137 .260 [-6.600, 1.818]
 sig_stat_data_PUTAMEN_R 0.903 (0.733) 1.232 .223 [-0.565, 2.372] 2.999
 groupSUB 6.773 (2.603) 2.602 .012 * [ 1.562, 11.983] 114.979
 sig_stat_data_DIENCEPHALON_VENTRAL_R -0.497 (0.752) -0.662 .511 [-2.002, 1.007] 3.898
 data_cluster1 -0.060 (0.124) -0.479 .634 [-0.308, 0.189] 2.525
 data_cluster2 0.322 (0.331) 0.973 .335 [-0.341, 0.985] 2.625
 age -0.043 (0.025) -1.710 .093 . [-0.093, 0.007] 1.084
 sex.x女 -0.092 (0.561) -0.165 .870 [-1.214, 1.030] 1.197
 sig_stat_data_PUTAMEN_R:groupSUB -2.436 (1.010) -2.412 .019 * [-4.458, -0.414] 2.265
 groupSUB:sig_stat_data_DIENCEPHALON_VENTRAL_R -0.431 (0.931) -0.463 .645 [-2.295, 1.433] 3.669
 groupSUB:data_cluster1 -0.167 (0.173) -0.967 .338 [-0.514, 0.179] 67.361
 groupSUB:data_cluster2 -0.078 (0.440) -0.177 .860 [-0.958, 0.803] 66.832
 ───────────────────────────────────────────────────────────────────────────────────────────────────────

 Standardized Coefficients (β):
 Outcome Variable: PC1
 N = 70
 ──────────────────────────────────────────────────────────────────────────────────────────────────────────────────
 β S.E. t p [95% CI of β] r(partial) r(part)
 ──────────────────────────────────────────────────────────────────────────────────────────────────────────────────
 sig_stat_data_PUTAMEN_R 0.114 (0.092) 1.232 .223 [-0.071, 0.299] 0.160 0.066
 groupSUB 1.488 (0.572) 2.602 .012 * [ 0.343, 2.633] 0.323 0.139
 sig_stat_data_DIENCEPHALON_VENTRAL_R -0.070 (0.105) -0.662 .511 [-0.280, 0.141] -0.087 -0.035
 data_cluster1 -0.041 (0.085) -0.479 .634 [-0.210, 0.129] -0.063 -0.026
 data_cluster2 0.084 (0.086) 0.973 .335 [-0.089, 0.257] 0.127 0.052
 age -0.095 (0.056) -1.710 .093 . [-0.206, 0.016] -0.219 -0.091
 sex.x女 -0.010 (0.058) -0.165 .870 [-0.126, 0.107] -0.022 -0.009
 sig_stat_data_PUTAMEN_R:groupSUB -0.194 (0.080) -2.412 .019 * [-0.354, -0.033] -0.302 -0.129
 groupSUB:sig_stat_data_DIENCEPHALON_VENTRAL_R -0.047 (0.102) -0.463 .645 [-0.252, 0.157] -0.061 -0.025
 groupSUB:data_cluster1 -0.423 (0.438) -0.967 .338 [-1.299, 0.453] -0.126 -0.052
 groupSUB:data_cluster2 -0.077 (0.436) -0.177 .860 [-0.950, 0.796] -0.023 -0.009
 ──────────────────────────────────────────────────────────────────────────────────────────────────────────────────

bruceR::PROCESS(new_data,y = "PC1",x="sig_stat_data_PUTAMEN_R",mods = "group",covs = c("age","sex.x"))

****************** PART 1. Regression Model Summary ******************

 PROCESS Model Code : 1 (Hayes, 2018; www.guilford.com/p/hayes3)
 PROCESS Model Type : Simple Moderation
 - Outcome (Y) : PC1
 - Predictor (X) : sig_stat_data_PUTAMEN_R
 - Mediators (M) : -
 - Moderators (W) : group
 - Covariates (C) : age, sex.x
 - HLM Clusters : -

 All numeric predictors have been grand-mean centered.
 (For details, please see the help page of PROCESS.)

 Formula of Outcome:
 - PC1 ~ age + sex.x + sig_stat_data_PUTAMEN_R*group

 CAUTION:
 Fixed effect (coef.) of a predictor involved in an interaction
 denotes its "simple effect/slope" at the other predictor = 0.
 Only when all predictors in an interaction are mean-centered
 can the fixed effect denote the "main effect"!

 Model Summary

 ────────────────────────────────────────────────────────
 (1) PC1 (2) PC1
 ────────────────────────────────────────────────────────
 (Intercept) -0.383 -2.552 ***
 (0.990) (0.551)
 age -0.075 -0.036
 (0.048) (0.025)
 sex.x女 0.406 0.009
 (1.020) (0.533)
 sig_stat_data_PUTAMEN_R -3.865 *** 0.828
 (0.837) (0.746)
 groupSUB 3.975 ***
 (0.296)
 sig_stat_data_PUTAMEN_R:groupSUB -2.241 *
 (1.023)
 ────────────────────────────────────────────────────────
 R^2 0.268 0.808
 Adj. R^2 0.235 0.793
 Num. obs. 70 70
 ────────────────────────────────────────────────────────
 Note. * p < .05, ** p < .01, *** p < .001.

 ************ PART 2. Mediation/Moderation Effect Estimate ************

 Package Use : ‘interactions’ (v1.1.5)
 Effect Type : Simple Moderation (Model 1)
 Sample Size : 70
 Random Seed : -
 Simulations : -

 Interaction Effect on "PC1" (Y)
 ───────────────────────────────────────────────────────
 F df1 df2 p
 ───────────────────────────────────────────────────────
 sig_stat_data_PUTAMEN_R * group 4.80 1 64 .032 *
 ───────────────────────────────────────────────────────

 Simple Slopes: "sig_stat_data_PUTAMEN_R" (X) ==> "PC1" (Y)
 ─────────────────────────────────────────────────────────
 "group" Effect S.E. t p [95% CI]
 ─────────────────────────────────────────────────────────
 CON 0.828 (0.746) 1.110 .271 [-0.662, 2.318]
 SUB -1.413 (0.698) -2.024 .047 * [-2.807, -0.019]
 ─────────────────────────────────────────────────────────

pc2_model <- lm(PC2~sig_stat_data_PUTAMEN_R*group+sig_stat_data_DIENCEPHALON_VENTRAL_R*group+
 data_cluster1*group+data_cluster2*group+age+sex.x,data=new_data)
GLM_summary(pc2_model)

General Linear Model (OLS Regression)

 Model Fit:
 F(11, 58) = 1.45, p = 0.174
 R² = 0.21624 (Adjusted R² = 0.06759)

 Unstandardized Coefficients:
 Outcome Variable: PC2
 N = 70
 ──────────────────────────────────────────────────────────────────────────────────────────────────────
 b S.E. t p [95% CI of b] VIF
 ──────────────────────────────────────────────────────────────────────────────────────────────────────
 (Intercept) -0.723 (2.073) -0.349 .729 [-4.872, 3.427]
 sig_stat_data_PUTAMEN_R -0.355 (0.723) -0.491 .625 [-1.802, 1.093] 2.999
 groupSUB -1.271 (2.567) -0.495 .622 [-6.408, 3.867] 114.979
 sig_stat_data_DIENCEPHALON_VENTRAL_R 0.179 (0.741) 0.241 .810 [-1.305, 1.662] 3.898
 data_cluster1 -0.032 (0.123) -0.265 .792 [-0.278, 0.213] 2.525
 data_cluster2 0.389 (0.327) 1.192 .238 [-0.265, 1.043] 2.625
 age -0.048 (0.025) -1.928 .059 . [-0.097, 0.002] 1.084
 sex.x女 0.635 (0.553) 1.149 .255 [-0.471, 1.742] 1.197
 sig_stat_data_PUTAMEN_R:groupSUB -0.082 (0.996) -0.083 .934 [-2.076, 1.911] 2.265
 groupSUB:sig_stat_data_DIENCEPHALON_VENTRAL_R 1.041 (0.918) 1.133 .262 [-0.797, 2.879] 3.669
 groupSUB:data_cluster1 -0.026 (0.171) -0.155 .878 [-0.368, 0.315] 67.361
 groupSUB:data_cluster2 0.286 (0.434) 0.659 .513 [-0.582, 1.153] 66.832
 ──────────────────────────────────────────────────────────────────────────────────────────────────────

 Standardized Coefficients (β):
 Outcome Variable: PC2
 N = 70
 ─────────────────────────────────────────────────────────────────────────────────────────────────────────────────
 β S.E. t p [95% CI of β] r(partial) r(part)
 ─────────────────────────────────────────────────────────────────────────────────────────────────────────────────
 sig_stat_data_PUTAMEN_R -0.099 (0.201) -0.491 .625 [-0.502, 0.304] -0.064 -0.057
 groupSUB -0.617 (1.246) -0.495 .622 [-3.112, 1.878] -0.065 -0.058
 sig_stat_data_DIENCEPHALON_VENTRAL_R 0.055 (0.230) 0.241 .810 [-0.404, 0.515] 0.032 0.028
 data_cluster1 -0.049 (0.185) -0.265 .792 [-0.419, 0.321] -0.035 -0.031
 data_cluster2 0.224 (0.188) 1.192 .238 [-0.153, 0.601] 0.155 0.139
 age -0.233 (0.121) -1.928 .059 . [-0.476, 0.009] -0.245 -0.224
 sex.x女 0.146 (0.127) 1.149 .255 [-0.108, 0.401] 0.149 0.134
 sig_stat_data_PUTAMEN_R:groupSUB -0.014 (0.175) -0.083 .934 [-0.365, 0.336] -0.011 -0.010
 groupSUB:sig_stat_data_DIENCEPHALON_VENTRAL_R 0.252 (0.223) 1.133 .262 [-0.193, 0.698] 0.147 0.132
 groupSUB:data_cluster1 -0.148 (0.954) -0.155 .878 [-2.057, 1.762] -0.020 -0.018
 groupSUB:data_cluster2 0.626 (0.950) 0.659 .513 [-1.276, 2.528] 0.086 0.077
 ─────────────────────────────────────────────────────────────────────────────────────────────────────────────────

pc1_model_sbm <- lm(PC1~sig_seed1_mean_dep*group+sig_seed2_mean_dep*group+age+sex.x,data=new_data)
GLM_summary(pc1_model_sbm)

General Linear Model (OLS Regression)

 Model Fit:
 F(7, 62) = 37.66, p = 5e-20 ***
 R² = 0.80958 (Adjusted R² = 0.78808)

 Unstandardized Coefficients:
 Outcome Variable: PC1
 N = 70
 ─────────────────────────────────────────────────────────────────────────────────────
 b S.E. t p [95% CI of b] VIF
 ─────────────────────────────────────────────────────────────────────────────────────
 (Intercept) -2.182 (2.173) -1.004 .319 [-6.525, 2.162]
 sig_seed1_mean_dep -0.034 (0.128) -0.269 .789 [-0.290, 0.221] 2.479
 groupSUB 6.507 (2.636) 2.468 .016 * [ 1.237, 11.777] 109.221
 sig_seed2_mean_dep 0.322 (0.344) 0.937 .352 [-0.365, 1.009] 2.615
 age -0.040 (0.026) -1.547 .127 [-0.092, 0.012] 1.072
 sex.x女 -0.368 (0.571) -0.645 .521 [-1.509, 0.772] 1.148
 sig_seed1_mean_dep:groupSUB -0.207 (0.178) -1.158 .251 [-0.563, 0.150] 66.254
 groupSUB:sig_seed2_mean_dep -0.017 (0.449) -0.038 .970 [-0.914, 0.879] 64.385
 ─────────────────────────────────────────────────────────────────────────────────────

 Standardized Coefficients (β):
 Outcome Variable: PC1
 N = 70
 ───────────────────────────────────────────────────────────────────────────────────────────────
 β S.E. t p [95% CI of β] r(partial) r(part)
 ───────────────────────────────────────────────────────────────────────────────────────────────
 sig_seed1_mean_dep -0.023 (0.087) -0.269 .789 [-0.198, 0.151] -0.034 -0.015
 groupSUB 1.430 (0.579) 2.468 .016 * [ 0.272, 2.587] 0.299 0.137
 sig_seed2_mean_dep 0.084 (0.090) 0.937 .352 [-0.095, 0.263] 0.118 0.052
 age -0.089 (0.057) -1.547 .127 [-0.204, 0.026] -0.193 -0.086
 sex.x女 -0.038 (0.059) -0.645 .521 [-0.157, 0.080] -0.082 -0.036
 sig_seed1_mean_dep:groupSUB -0.522 (0.451) -1.158 .251 [-1.424, 0.379] -0.146 -0.064
 groupSUB:sig_seed2_mean_dep -0.017 (0.445) -0.038 .970 [-0.906, 0.872] -0.005 -0.002
 ───────────────────────────────────────────────────────────────────────────────────────────────

pc2_model_sbm <- lm(PC2~sig_seed1_mean_dep*group+sig_seed2_mean_dep*group+age+sex.x,data=new_data)
GLM_summary(pc2_model_sbm)

General Linear Model (OLS Regression)

 Model Fit:
 F(7, 62) = 1.40, p = 0.223
 R² = 0.13621 (Adjusted R² = 0.03868)

 Unstandardized Coefficients:
 Outcome Variable: PC2
 N = 70
 ────────────────────────────────────────────────────────────────────────────────────
 b S.E. t p [95% CI of b] VIF
 ────────────────────────────────────────────────────────────────────────────────────
 (Intercept) -0.896 (2.093) -0.428 .670 [-5.080, 3.289]
 sig_seed1_mean_dep -0.037 (0.123) -0.298 .767 [-0.283, 0.210] 2.479
 groupSUB -0.045 (2.540) -0.018 .986 [-5.122, 5.032] 109.221
 sig_seed2_mean_dep 0.384 (0.331) 1.162 .250 [-0.277, 1.046] 2.615
 age -0.044 (0.025) -1.763 .083 . [-0.094, 0.006] 1.072
 sex.x女 0.671 (0.550) 1.220 .227 [-0.428, 1.769] 1.148
 sig_seed1_mean_dep:groupSUB -0.029 (0.172) -0.167 .868 [-0.372, 0.315] 66.254
 groupSUB:sig_seed2_mean_dep 0.097 (0.432) 0.225 .823 [-0.767, 0.961] 64.385
 ────────────────────────────────────────────────────────────────────────────────────

 Standardized Coefficients (β):
 Outcome Variable: PC2
 N = 70
 ───────────────────────────────────────────────────────────────────────────────────────────────
 β S.E. t p [95% CI of β] r(partial) r(part)
 ───────────────────────────────────────────────────────────────────────────────────────────────
 sig_seed1_mean_dep -0.055 (0.186) -0.298 .767 [-0.427, 0.316] -0.038 -0.035
 groupSUB -0.022 (1.234) -0.018 .986 [-2.488, 2.444] -0.002 -0.002
 sig_seed2_mean_dep 0.222 (0.191) 1.162 .250 [-0.160, 0.603] 0.146 0.137
 age -0.215 (0.122) -1.763 .083 . [-0.460, 0.029] -0.218 -0.208
 sex.x女 0.154 (0.126) 1.220 .227 [-0.099, 0.407] 0.153 0.144
 sig_seed1_mean_dep:groupSUB -0.161 (0.961) -0.167 .868 [-2.081, 1.760] -0.021 -0.020
 groupSUB:sig_seed2_mean_dep 0.213 (0.947) 0.225 .823 [-1.681, 2.106] 0.029 0.027
 ───────────────────────────────────────────────────────────────────────────────────────────────
